# An Early Th1 Response Is a Key Factor for a Favorable COVID-19 Evolution

**DOI:** 10.3390/biomedicines10020296

**Published:** 2022-01-27

**Authors:** Francisco Javier Gil-Etayo, Sara Garcinuño, Alberto Utrero-Rico, Oscar Cabrera-Marante, Daniel Arroyo-Sanchez, Esther Mancebo, Daniel Enrique Pleguezuelo, Edgard Rodríguez-Frías, Luis M. Allende, Pablo Morales-Pérez, María José Castro-Panete, Antonio Lalueza, Carlos Lumbreras, Estela Paz-Artal, Antonio Serrano

**Affiliations:** 1 Department of Immunology, Hospital Universitario 12 de Octubre, 28041 Madrid, Spain; fgile@salud.madrid.org (F.J.G.-E.); oscar.cabrera@salud.madrid.org (O.C.-M.); darroyos@salud.madrid.org (D.A.-S.); esther.mancebo@salud.madrid.org (E.M.); dpleguezuelo@salud.madrid.org (D.E.P.); edgardalfonso.rodriguezde@salud.madrid.org (E.R.-F.); luis.allende@salud.madrid.org (L.M.A.); pmorales@h12o.es (P.M.-P.); mariajose.castro@salud.madrid.org (M.J.C.-P.); estela.paz@salud.madrid.org (E.P.-A.); 2 Instituto de Investigación Sanitaria Hospital 12 de Octubre (imas12), 28041 Madrid, Spain; garcinunosara@gmail.com (S.G.); autrero.imas12@h12o.es (A.U.-R.); antonio.lalueza@salud.madrid.org (A.L.); carlos.lumbreras@salud.madrid.org (C.L.); 3 Department of Immunology, Ophthalmology and Otorhinolaryngology, Facultad de Medicina, Universidad Complutense de Madrid, 28040 Madrid, Spain; 4 Department of Internal Medicine, Hospital Universitario 12 de Octubre, 28041 Madrid, Spain; 5 Department of Medicine, Facultad de Medicina, Universidad Complutense de Madrid, 28040 Madrid, Spain; 6 Biomedical Research Centre Network for Epidemiology and Public Health (CIBERESP), 28029 Madrid, Spain

**Keywords:** COVID-19, Th1, T helper, cell mediated immunity, imbalanced immune response, Th17, Th2, T-cell subsets, SARS-CoV2

## Abstract

The Th1/Th2 balance plays a crucial role in the progression of different pathologies and is a determining factor in the evolution of infectious diseases. This work has aimed to evaluate the early, or on diagnosis, T-cell compartment response, T-helper subsets and anti-SARS-CoV-2 antibody specificity in COVID-19 patients and to classify them according to evolution based on infection severity. A unicenter, randomized group of 146 COVID-19 patients was divided into four groups in accordance with the most critical events during the course of disease. The immunophenotype and T-helper subsets were analyzed by flow cytometry. Asymptomatic SARS-CoV-2 infected individuals showed a potent and robust Th1 immunity, with a lower Th17 and less activated T-cells at the time of sample acquisition compared not only with symptomatic patients, but also with healthy controls. Conversely, severe COVID-19 patients presented with Th17-skewed immunity, fewer Th1 responses and more activated T-cells. The multivariate analysis of the immunological and inflammatory parameters, together with the comorbidities, showed that the Th1 response was an independent protective factor for the prevention of hospitalization (OR 0.17, 95% CI 0.03–0.81), with an AUC of 0.844. Likewise, the Th1 response was found to be an independent protective factor for severe forms of the disease (OR 0.09, 95% CI: 0.01–0.63, *p* = 0.015, AUC: 0.873). In conclusion, a predominant Th1 immune response in the acute phase of the SARS-CoV-2 infection could be used as a tool to identify patients who might have a good disease evolution.

## 1. Introduction

The SARS-CoV-2 infection is characterized by a wide spectrum of clinical profiles: an asymptomatic process, mild coronavirus disease (COVID-19) (with or without need for hospitalization) and severe COVID-19 with complications that can lead to the death of the patient [1]. Several characteristics such as male gender, genetic susceptibility, age, comorbidities and coagulant abnormalities have been associated with a higher risk of disease severity although the exact mechanisms that contribute to the fact that some patients evolve less aggressively have not been clearly defined [2,3,4,5,6,7].

The immune response mounted against a particular infection is a determining factor for the disease outcome [8,9]. This phenomenon has been widely studied in diseases like leprosy and Human Immunodeficiency Virus (HIV) infection, in which a T-helper 1 (Th1) response is associated with a better prognosis whereas the Th2 response is associated with the development of aggressive forms of the disease [9,10,11,12,13].

The signals through their CD4+ T cells are differentiated into Th1 or Th2 in an infectious process. Their differentiation depends on the specific recognition of the antigen, co-stimulatory signals and the cytokine context [14]. Most of the cytokines involved in this process are produced by cells of the innate immune system. The differentiation of Th1 cells is produced thanks to the release of IL-12, mainly by antigen-presenting cells. IL-12 signals, through their specific receptor IL-12R, are capable of triggering an intracellular signaling cascade that results in the activation of a crucial transcription factor for Th1 differentiation, T-bet [15,16,17,18,19]. On the other hand, the Th2 differentiation process is driven by IL-4 [20,21]. IL-4 is a cytokine produced by mast cells against helminths or allergic reactions [22]. In fact, one of the greatest producers of IL-4 are Th2 cells, so the Th2 cells also contribute to their own differentiation in a paracrine way. When this cytokine binds to its specific receptor, IL-4R, it triggers an intracellular signaling cascade, which causes an augmented expression of GATA3, an essential transcription factor for Th2 differentiation [23,24].

Poor evolution of the COVID-19 patient has been associated with relevant abnormalities of the immune system function [25,26,27]. Severe COVID-19 patients present immune dysfunctions, including severe lymphopenia with an augmented activation of the T-cell compartment [28,29,30] and an incorrect Th polarization [31,32]. Furthermore, innate immunity is also involved in profound dysregulation of monocytes, dendritic cells, Natural Killer (NK) cells and Mucosal-associated Invariant T cells [33,34,35,36].

The most life-threatening immune complication of the disease is the huge cytokine production called “cytokine storm.” This suggests that there is an additional effort to clear the infection after the claudicating of the immune system. However, the lack of prompt treatment regarding this scenario could result in multiorgan failure and death [37,38,39].

The greatest efforts within the health care system have been directed towards the study of the severity parameters in hospitalized COVID-19 patients, including death and Intensive Care Unit (ICU) admission requirements. Nevertheless, little is known about the differential characteristics of COVID-19 patients who do not require hospitalization. As occurs in severe cases of the disease, the immune system could present a potential benefit in the management of COVID-19 patients.

This study has aimed to evaluate the T-cell compartment response, Th subsets and specificity of anti-SARS-CoV-2 antibodies in COVID-19 patients according to infection severity and to obtain more information about the pathophysiology of the disease.

## 2. Materials and Methods

### 2.1. Study Design

A prospective observational study that recruited patients at early stages of the COVID-19 pandemic was conducted in a Spanish tertiary university hospital.

Immunophenotypes of peripheral T lymphocytes and anti-SARS-CoV-2 antibodies were evaluated at the time of diagnosis. Hospitalized patients were followed-up until discharge or death. Clinical information about non-hospitalized patients was obtained in the Emergency Department.

### 2.2. Patients

A randomized cohort of 160 COVID-19 patients was enrolled in the Emergency Department of the Hospital Universitario 12 de Octubre (Madrid, Spain) from 9 March to 29 April 2021. Inclusion criteria were: (1) Adult patients (>18 years) with high suspicion of COVID-19 infection; (2) confirmed diagnosis by RT-PCR; (3) Ddagnosis made in the early acute phase of the disease; (4) follow-up until discharge or death; and (5) non-vaccinated COVID-19 patients.

Fourteen patients were excluded as they were lost to the follow-up. Finally, 146 COVID-19 patients were enrolled in the study.

A group made up of 29 anonymous blood donors (negative for SARS-CoV-2 tests) with a median age of 50 (IQR: 43.75–59.25) was created to compare T lymphocytes immunophenotypes with COVID-19 patients. This group was created during the first wave of the pandemic, before vaccination was available.

### 2.3. Patient Classification

The COVID-19 patients (146) were divided by requirement for hospitalization: (1) Non-hospitalized: *N* = 54; and (2) hospitalized: *N* = 92. Non-hospitalized patients were also classified according to the presence of COVID-19 symptoms: (1) Asymptomatic: *N* = 12 and (2) Symptomatic: *N* = 42.

**Figure 1:**
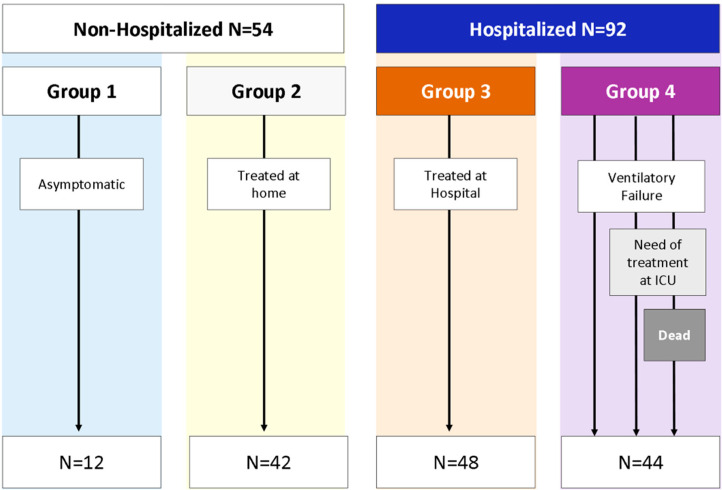
Classification of patients according to the most critical event during the evolution of the disease. (Group 1) Non-Hospitalized patients who did not develop symptoms. (Group 2) Non-Hospitalized patients who developed mild symptoms. (Group 3) Hospitalized patients with mild to moderate symptoms. (Group 4) Hospitalized patients with ARDS.

Four groups of COVID-19 patients were created in accordance with the disease progression: (1) Asymptomatic (*N* = 12); (2) symptomatic, who did not require hospital admission (*N* = 42); (3) mild to moderate hospitalized patients, who did not develop acute respiratory distress syndrome (ARDS) (*N* = 48); and (4) COVID-19 patients who developed ARDS (*N* = 44) (Figure 1).

### 2.4. Study Definitions

COVID-19 case was defined as a positive result for SARS-CoV-2 reverse transcription polymerase chain reaction (RT-PCR) assay performed on a nasal swab sample from patients in whom COVID-19 was suspected.

Ventilatory failure was defined as a SaO_2_/FiO_2_ < 300 (blood oxygen pressure/fractional inspired oxygen), or the need for mechanical ventilation (either non-invasive positive pressure ventilation or invasive mechanical ventilation).

Poor outcome was defined as the presence of at least one of the following criteria: (a) ventilatory failure; (b) admission to the intensive care unit (ICU); or (c) death during admission by any cause.

Lymphopenia was defined as a total lymphocyte count of less than 0.85 cells/mL.

Comorbidities are medical conditions associated with a higher risk of becoming severely ill from COVID-19 [40]. Patients with comorbidities were considered to be those with a history of diabetes mellitus, obesity, arterial hypertension, dyslipidemia, acute myocardial infarction, advanced chronic kidney disease, active smoking or ex-smokers. Association of the presence of comorbidities with the evolution was studied jointly (any comorbidity) and also individually for each condition.

Advanced chronic kidney disease is defined as a glomerular filtration rate less than 30 mL/min. It includes stages 4 and 5 of Chronic kidney disease as defined in the K/DOQI clinical practice guidelines for chronic kidney disease [41].

### 2.5. Data Collection

An anonymized database was created. It contained the patient data, including demographic, clinical and laboratory data from the electronic medical record. Laboratory parameters included D-dimer (DD), lactate dehydrogenase (LDH), C reactive protein (CRP) and the number of lymphocytes.

### 2.6. Samples

Plasma and EDTA-treated blood samples were collected in the first 24h after the admission in the Emergency Department with a median of 7 days from the onset of the symptoms in the case of the symptomatic COVID-19 patients.

### 2.7. T-Cell Subsets

EDTA-treated whole blood was incubated using the corresponding monoclonal antibodies: anti-CCR7-FITC, anti-CD57-PE, anti-CD3-PerCP5.5, anti-CD45RA-PCy7 and anti-CD8-APC (all from BDBiosciences, New York, NY, USA); anti-CD4-APC-AlexaFluor750 and anti-HLA-DR-PB (all from Beckman Coulter, Miami, FL, USA). Proportions of T-cells were analyzed by flow cytometry using a Navios Cytometer and Kaluza Software (Beckman Coulter, Miami, FL, USA) [40].

### 2.8. The Subsets

EDTA-treated whole blood was incubated using the corresponding monoclonal antibodies: anti-CCR6-PB, anti-PD1-PCy7, anti-CD3-PerCP5.5, anti-CXCR3-PE and anti-ICOS-APCH7 (all from BDBiosciences, New York, NY, USA); anti-CD4-KO (from Beckman Coulter, Miami, FL, USA). Proportions of Th subsets were analyzed by flow cytometry using a Navios Cytometer and Kaluza Software (Beckman Coulter, Miami, FL, USA).

According to the expression of cell surface markers in CD4+ T cells, they could be classified as: Th1 cells (CXCR3+/CCR6-), Th17 cells (CXCR3-/CCR6+) and Th2 cells (CXCR3-/CCR6-). Regarding the activation status, each one could be divided into quiescent cells (ICOS-/PD1-), early activated cells (ICOS+/PD-1-), late activated cells (ICOS+/PD-1+) and exhausted or senescent cells (ICOS-/PD-1+) [32].

### 2.9. Evaluation of Anti-SARS-CoV-2 Antibodies

The Multiplex addressable laser bead immunoassay BioPlex^®^2200 SARS-CoV-2 IgG Panel (Bio-Rad, Hercules, CA, USA) was used to detect the presence of IgG antibodies against the SARS-CoV-2 antigens in plasma: spike 1 (S1), spike 2 (S2), receptor-binding domain (RBD) and nucleocapsid (NCap). The manufacturer’s recommended cutoff was employed.

### 2.10. Statistical Analysis

The results in the discrete variables were shown as a frequency and a percentage. Comparisons were made with Pearson’s Chi-square test (or Fisher’s exact test). Odds ratio represents the relative measure of an effect.

Results of the continuous variables were expressed as median accompanied by the interquartile range in brackets. Mann–Whitney U test and Kruskal–Wallis test were used for comparisons.

Multivariate analyses were performed through a logistic regression model using variables that had presented a *p*-value < 0.12 in a previous univariate analysis.

Center-scaled variables were used for hierarchical clustering analysis and heatmap representation using ComplexHeatmap R package.

Probabilities under 0.05 were considered significant. Data were analyzed with MedCalc for Windows version 19.3 (MedCalc Software, Ostend, Belgium).

## 3. Results

### 3.1. Population Characteristics and Biochemical Markers

Median age of the COVID-19 patients was 56 (43–69.5) years without significant differences in the proportion of men and women (56.5% men). No significant differences were found between COVID-19 patients and healthy controls in relation to age and gender (data not shown).

**Figure 2:**
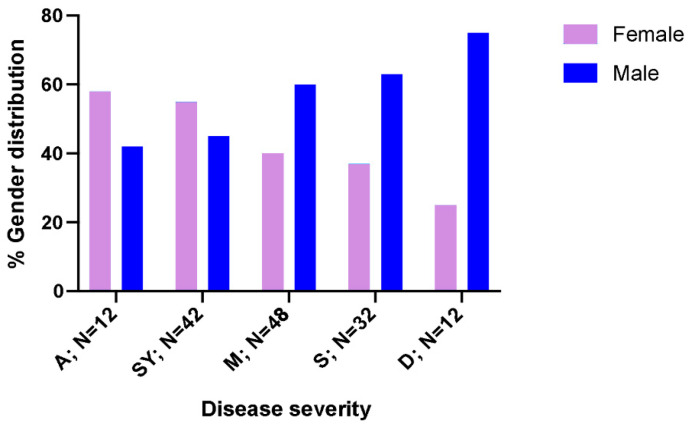
Sex distribution according to disease progression. A: asymptomatic; SY: symptomatic without hospitalization requirements; M: mild to moderate; S: severe; D: deceased patients. *p*-value = 0.021. Light purple: Female; Dark blue: Male.

Once COVID-19 patients were divided according to hospitalization requirements, we observed that non-hospitalized patients were significantly younger than hospitalized patients: median 48.5 years vs. 58.5 (*p* = 0.004). Gender distribution according to hospitalization requirements showed that males presented higher rates of hospitalization compared to females (63% men, *p* = 0.038) (Table 1). However, it should be mentioned that the biggest proportion of men was found in patients with severe disease (Figure 2), especially in deceased patients (75% men, *p* = 0.021).

The study of the inflammatory parameters (Table 1) showed the differences between non-hospitalized and hospitalized COVID-19 patients. Patients who fulfilled hospitalization requirements had higher median levels of LDH: 336 U/L (225.5–402.2) vs. 276.5 (222.5–310) (*p* = 0.001), CRP: 6 mg/dL (3.7–11) vs. 2.9 (1.3–5.2) (*p* < 0.001). DD levels were higher in hospitalized patients, but these differences were not significant compared to non-hospitalized patients: DD: 691 ng/mL (410–1414) vs. 555 (299.5–907.5) (*p* = 0.058). However, the comparison of DD was biased, as the number of patients in whom DD levels were evaluated as low as DDs were only tested in those patients with a poor clinical profile prognosis.

No cases of this complication were observed in non-hospitalized patients when ARDS rates were studied.

### 3.2. Specificity of Anti-SARS-CoV-2 Antibodies

A total of 75 (51.7%) patients developed a positive antibody response with the presence of at least one type of the tested IgG antibody against SARS-CoV-2 virus in the early stage of the disease. Non-hospitalized patients presented similar positive rates compared to hospitalized patients (51% vs. 45.6%; *p* = 0.076). Hence, the association between hospitalization requirements and humoral response did not show any statistical significance (Table S1).

Similarly, the production of anti-SARS-CoV-2 antibodies in asymptomatic patients was evaluated against symptomatic and severe patients, respectively. The rates of production of antibodies were similar in both comparisons (data not shown).

### 3.3. CD4 and CD8 Subpopulations and the Severity of the Disease

COVID-19 patients had profound lymphopenia compared with healthy controls, including the median total number of lymphocytes: 1100 (800–1500) vs. 1846 (1585–2129) cells/µL (*p* < 0.001), the CD4+ T cells: 636 (416–708) vs. 783 (664–1099) cells/µL (*p* = 0.012) and CD8+ T cells: 296 (185–477) vs. 404 (285–524) cells/µL (*p* = 0.022) (Table 2).

**Figure 3:**
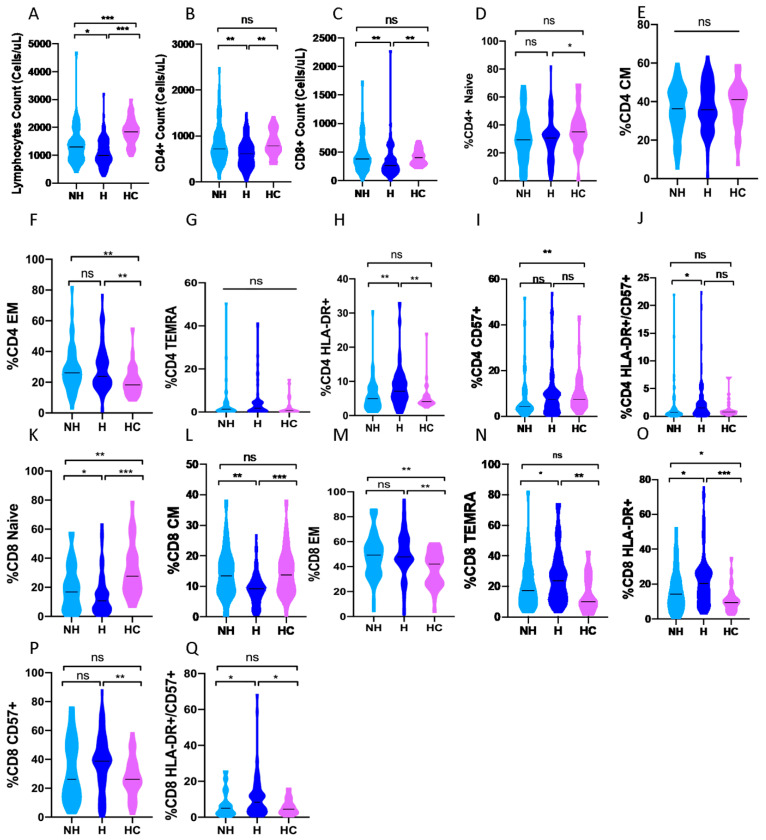
Differences in lymphocyte subsets between healthy controls, non-hospitalized and hospitalized COVID-19 patients separately. (**A**) Lymphocyte count (cells/µL) (**B**) Number of CD4+ (cells/µL) T cells (**C**) Number of CD8+ (cells/µL) T cells (**D**) Percentage of naive (CCR7+/CD45RA+) CD4+ T cells (**E**) Percentage of CM (central memory, CCR7+/CD45RA-) CD4+ T cells (**F**) Percentage of EM (effector memory, CCR7-/CD45RA-) CD4+ T cells (**G**) Percentage of TEMRA (Terminal differentiated T memory cells, CCR7-/CD45RA+) CD4+ T cells (**H**) Percentage of activated (HLA-DR+) CD4+ T cells (**I**) Percentage of senescent (CD57+) CD4+ T cells (**J**) Percentage of double positive (HLA-DR+/CD57+) CD4+ T cells (**K**) Percentage of naive (CCR7+/CD45RA+) CD8+ T cells (**L**) Percentage of CM (central memory, CCR7+/CD45RA-) CD8+ T cells (**M**) Percentage of EM (effector memory, CCR7-/CD45RA-) CD8+ T cells (**N**) Percentage of TEMRA (Terminal differentiated T memory cells, CCR7-/CD45RA+) CD8+ T cells (**O**) Percentage of activated (HLA-DR+) CD8+ T cells (**P**) Percentage of senescent (CD57+) CD8+ T cells (**Q**) Percentage of double positive (HLA-DR+/CD57+) CD8+ T cells. NH; Non-hospitalized COVID-19 patients, H; Hospitalized COVID-19 patients, HC; Healthy controls. ns, not-significant; * *p* < 0.05; ** *p* < 0.01; *** *p* < 0.001.

When our COVID-19 cohort was divided according to hospitalization requirements into non-hospitalized patients (*n* = 54) compared with hospitalized (*n* = 92) ones (Figure 1), non-hospitalized patients showed a higher median of total lymphocyte counts: 1300 (900–1600) vs. 1000 (800–1497) cells/µL, *p* = 0.012), CD4+ T cells: 718 (500–1026) vs. 610 (378-812) cells/µL (*p* = 0.004) and CD8+ T cells: 382 (238-558) vs. 266 (171–397) cells/µL (*p* = 0.003, Figure 3A–C).

**Figure 4:**
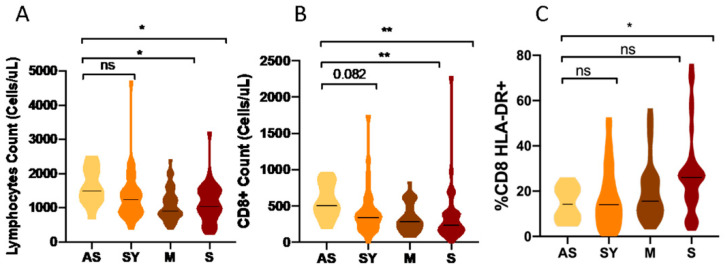
Lymphocytic comparison of asymptomatic COVID-19 patients vs. symptomatic ones. (**A**) Lymphocyte count (cells/µL) (**B**) Number of CD8+ (cells/µL) T cells (**C**) Percentage of activated (HLA-DR+) CD8+ T cells. AS; asymptomatic COVID-19 patients, SY; symptomatic COVID-19 patients without hospitalization requirements, M; mild to moderate hospitalized COVID-19 patients and S; Severe COVID-19 patients. ns, not-significant; * *p* < 0.05; ** *p* < 0.01.

We did not find significant differences in the number of lymphocytes or CD4+ T cells between asymptomatic patients (*n* = 12) and symptomatic patients who did not require hospitalization (*n* = 42) (Figure S1). However, asymptomatic patients showed a higher median of total lymphocyte counts (Figure 4A) compared with mild to moderate hospitalized patients (*N* = 48) and severe COVID-19 patients who developed ADRS (*N* = 44): 1500 (1200–2075), 915 (800–1300) and 1050 (789–1500) cells/µL, respectively (*p* = 0.011 and *p* = 0.043, respectively) when the comparison was performed according to disease severity.

Furthermore, the analysis of the median number of CD8+ T cells revealed that asymptomatic patients had a higher number of CD8+ T cells: 504 (402–816) cells/µL compared to non-hospitalized patients with symptoms: 343 (222–495) cells/µL (*p* = 0.017), non-severe hospitalized patients: 281 (185–462) cells/µL (*p* = 0.007), and severe hospitalized patients: 234 (145–392) cells/µL (*p* = 0.002), Figure S1C and Figure 4B.

Studying CD4+ and CD8+ T-cell compartments in depth, COVID-19 patients compared with healthy controls showed a significant reduction in the median percentage of naïve CD4+ T cells (CCR7+/CD45RA+): 30.3% (18.8–38.1) vs. 35% (29.3–46.6) (*p* < 0.001) and CD8+ T cells: 13% (5.5–13) vs. 27.6% (17.8–41.1) (*p* = 0.028, Table 2). These decreases in the proportion of CD8+ naïve T cells were also found in asymptomatic COVID-19 vs. healthy controls: median 12% (3.8–24.5) vs. 27.6% (17.8–41.1) (*p* = 0.005, Table S3) and in hospitalized COVID-19 patients versus non-hospitalized patients: median 10.7% (5.5–19) vs. 16.8% (6.7–32) (*p* = 0.043, Figure 3K).

The memory status of COVID-19 patients showed a significantly decreased median in the proportion of central memory (CM, CCR7+/CD45RA-) CD8+ T-cells compared to healthy controls: 8.3% (5.1–11.2) vs. 13.7% (8.4–18.7) (*p* < 0.001, Table 2). This same pattern of CD8+ T cells was observed when asymptomatic patients were compared to healthy controls: 5.9% (3.2–7.6) vs. 13.7% (8.4–18.7) (*p* < 0.001, Table S3).

In addition, the proportion of some effector phenotype subpopulations showed an augmentation in COVID-19 patients compared to healthy controls, including the effector memory (EM, CCR7-/CD45RA-) phenotype in both CD4+: 25.8% (18.6–35.2) vs. 18.3% (13.6–24.9)(*p* = 0.001) and CD8+ T-cells: 48.1% (40–60.2) vs. 42% (26.6–49.9) (*p* < 0.001, Table 2) and terminal differentiated effector memory re-expressing CD45RA (TEMRA, CCR7-/CD45RA+) CD8+ cells: 20.5% (11–30.5) vs. 10% (7.5–24.5) (*p* = 0.003, Table 2). These TEMRA CD8+ T-cells were also elevated in asymptomatic COVID-19 patients compared to healthy controls: 25% (17–33.5) vs. 10% (7.5–24.5) (*p* = 0.031, Table S3) and to symptomatic patients who did not require hospital admission: 25% (17–33.4) vs. 14.4% (9.3–26.4) (*p* = 0.08, Figure S1N).

The activation phenotype of T-cells showed that COVID-19 patients presented a higher proportion (median) of activated (HLA-DR+) CD4+ T cells: 6.2% (3.5–9.5) vs. 4.1% (3.5–5.5) (*p* = 0.023) and CD8+ T cells: 18% (9.1–26) vs. 9.5% (6–12) (*p* < 0.001, Table 2) compared to healthy controls. The same trend was observed when hospitalized patients were compared to non-hospitalized COVID-19 patients: 7.1% (4.7–10.5) vs. 5% (2.9–8) (*p* = 0.001 for CD4+, Figure 3H) and 20.4% (11.9–27.6) vs. 14.1% (7.9–22.6), (*p* = 0.012 for CD8+, Figure 3O). However, asymptomatic COVID-19 patients presented diminished cytotoxic activated status compared to severe patients: 16.5% (8.7–21.3) vs. 26% (16–29) (*p* = 0.04, Figure 4C) when the analysis was performed according to the severity of the disease.

Another population, that of senescent (CD57+) T-cells, was analyzed with the activation phenotype. In this case, COVID-19 patients showed a higher median in the proportion of senescent CD8+ cells compared to healthy controls: 38.7% (18.8–50) vs. 26.2% (20.4–35.9) (*p* = 0.024, Table 2). Furthermore, the comparison between asymptomatic patients and healthy controls showed similar results: 45.6% (21.1–63.1) vs. 26.2% (20–35.9) (*p* = 0.048, Table S3).

When we analyzed the concomitant expression of HLA-DR and CD57 between hospitalized and non-hospitalized COVID-19 patients, we observed a higher median proportion of double positive cells in hospitalized patients in both CD4+: 1.5% (0.5–2.6) vs. 0.7% (0.3–1.5) (*p* = 0.007, Figure 3J) and CD8+: 8.35% (3.4–13.7) vs. 5% (2–8) (*p* = 0.02, Figure 3Q).

No significant results were found regarding COVID-19 vs. healthy controls (Table 2), hospitalized vs. non-hospitalized (Figure 3), asymptomatic vs. symptomatic patients who did not require hospital admission (Figure S1), asymptomatic vs. healthy controls (Table S3) and asymptomatic patients vs. COVID-19 patients grouped according to disease severity (Figure S2 for CD4+ and Figure S3 for CD8+).

### 3.4. Th1, Th2 and Th17 Subsets in COVID-19 Patients and Healthy Controls

When the Th polarization of CD4+ T-cells was studied, COVID-19 patients compared to healthy controls showed a decreased median in the proportion of Th1: 4.8% (3.3–6.5) vs. 6.4% (4.9–8.2) (*p* = 0.011) and Th17: 4% (3.1–5.7) vs. 7% (3–10.6) (*p* = 0.018). COVID-19 patients also showed a decreased median proportion of early-activated Th1 (ICOS+/PD-1-): 0.3% (0.2–0.6) vs. 1.7% (0.7–2.1) (*p* < 0.001), Th2: 0.4% (0.2–0.8) vs. 2.81% (1.3–3.6) (*p* < 0.001) and Th17: 0.2% (0.1–0.4) vs. 1.2% (0.6–1.8) (*p* < 0.001). In addition, infected patients showed a lower median percentage of late-activated Th2 (ICOS+/PD-1+): 0.3% (0.2–0.5) vs. 1.2% (1.5–1.8) (*p* < 0.001) and Th17 cells: 0.2% (0.1–0.3) vs. 1.4% (0.6–2.1) (*p* < 0.001).

The median proportion of senescent cells (ICOS-/PD-1+) in patients (presumably generated after rapid activation and exhaustion) when compared with controls was lower in Th2 cells: 4.3% (2.1–7.7) vs. 11.5% (7.8–15.2) (*p* < 0.001) and Th17 cells: 2.1% (1.4–3.2) vs. 3.2% (1.6–7) (*p* = 0.036) and higher in Th1 cells: 7% (4.5–9.8) vs. 4.2% (3–7.7) (*p* = 0.008). Non-significant differences were found when quiescent cells (ICOS-/PD1-) were compared. Comparisons between Th polarization in patients and controls are described in Table S2.

**Figure 5:**
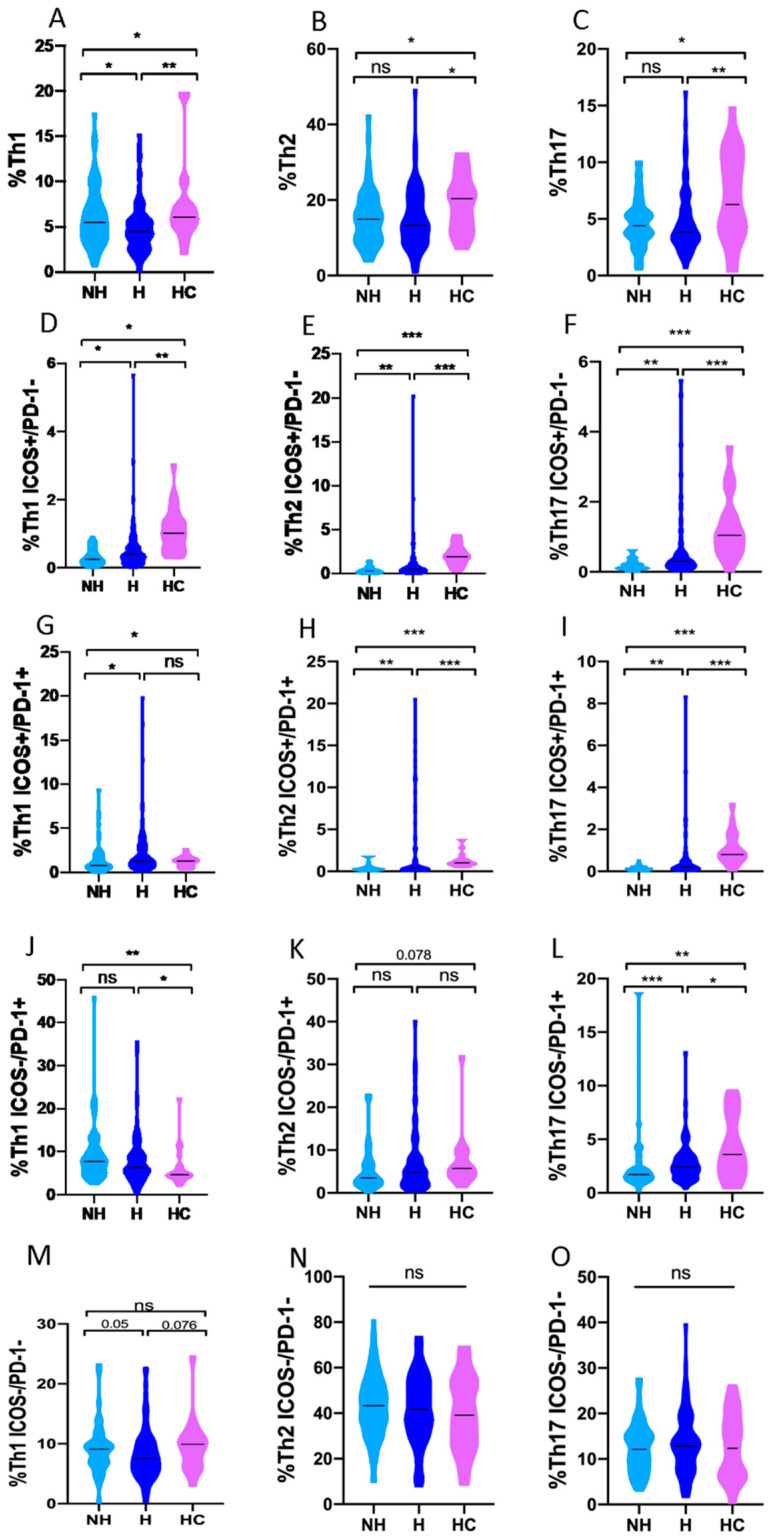
Differences of Th subsets between healthy controls, non-hospitalized and hospitalized COVID-19 patients separately. (**A**) Total percentage of Th1 CD4+ T cells (**B**) Total parentage of Th2 TCD4+ T cells (**C**) Total percentage of Th17 CD4+ T cells (**D**) Percentage of early-activated (ICOS+/PD-1-)Th1 CD4+ T cells (**E**) Percentage of early-activated (ICOS+/PD-1-)Th2 CD4+ T cells (**F**) Percentage of early-activated (ICOS+/PD-1-)Th17 CD4+ T cells (**G**) Percentage of late-activated (ICOS+/PD-1+)Th1 CD4+ T cells (**H**) Percentage of late-activated (ICOS+/PD-1+)Th2 CD4+ T cells (**I**) Percentage of late-activated (ICOS+/PD-1+)Th17 CD4+ T cells (**J**) Percentage of senescent (ICOS-/PD-1+)Th1 CD4+ T cells (**K**) Percentage of senescent (ICOS-/PD-1+)Th2 CD4+ T cells (**L**) Percentage of senescent (ICOS-/PD-1+)Th17 CD4+ T cells (**M**) Percentage of quiescent (ICOS-/PD-1-)Th1 CD4+ T cells (**N**) Percentage of quiescent (ICOS-/PD-1-)Th2 CD4+ T cells (**O**) Percentage of quiescent (ICOS-/PD-1-)Th17 CD4+ T cells. NH; Non-hospitalized COVID-19 patients, H; Hospitalized COVID-19 patients, HC; Healthy controls. ns, not significant; * *p* < 0.05; ** *p* < 0.01; *** *p* < 0.001.

The comparison of the Th response between healthy controls and non-hospitalized and hospitalized patients, respectively, is shown in Figure 5.

### 3.5. Th1, Th2 and Th17 Subsets in Non-Hospitalized and Hospitalized COVID-19 Patients

**Figure 6:**
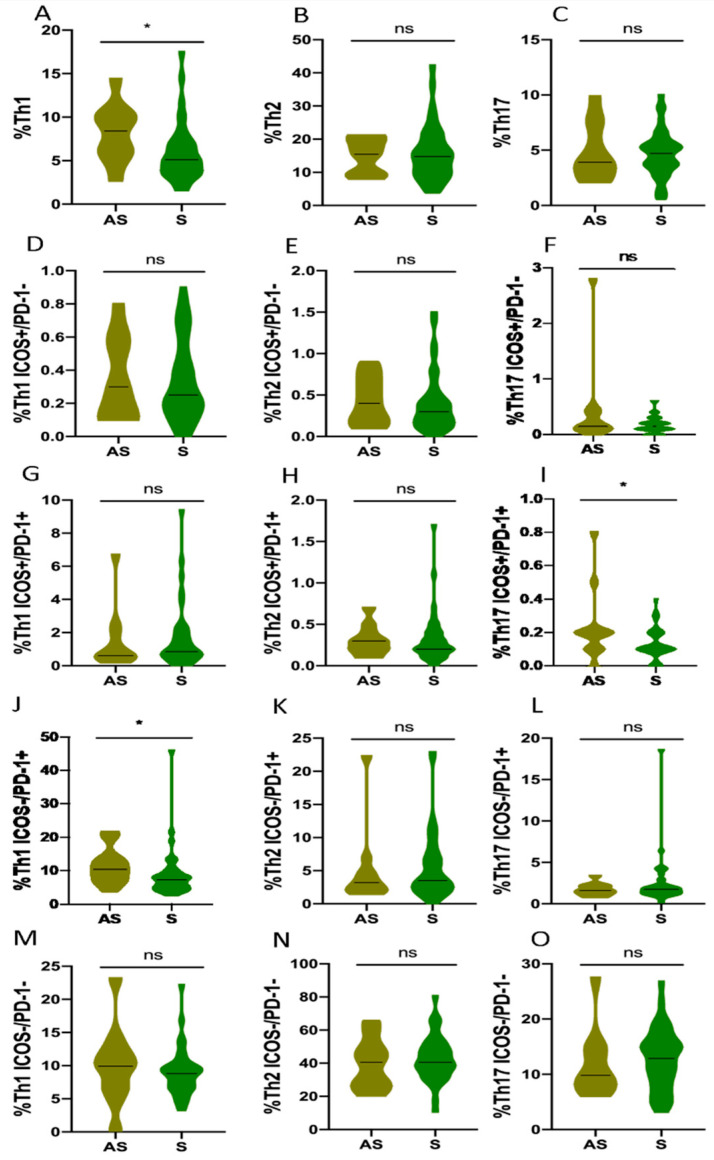
Th subsets in asymptomatic and symptomatic COVID-19 patients without hospitalization requirements. (**A**) Total percentage of Th1 CD4+ T cells (**B**) Total parentage of Th2 CD4+ T cells (**C**) Total percentage of Th17 CD4+ T cells (**D**) Percentage of early-activated (ICOS+/PD-1-)Th1 CD4+ T cells (**E**) Percentage of early-activated (ICOS+/PD-1-)Th2 CD4+ T cells (**F**) Percentage of early-activated (ICOS+/PD-1-)Th17 CD4+ T cells (**G**) Percentage of late-activated (ICOS+/PD-1+)Th1 CD4+ T cells (**H**) Percentage of late-activated (ICOS+/PD-1+)Th2 CD4+ T cells (**I**) Percentage of late-activated (ICOS+/PD-1+)Th17 CD4+ T cells (**J**) Percentage of senescent (ICOS-/PD-1+)Th1 CD4+ T cells (**K**) Percentage of senescent (ICOS-/PD-1+)Th2 CD4+ T cells (**L**) Percentage of senescent (ICOS-/PD-1+)Th17 CD4+ T cells (**M**) Percentage of quiescent (ICOS-/PD-1-)Th1 CD4+ T cells (**N**) Percentage of quiescent (ICOS-/PD-1-)Th2 CD4+ T cells (**O**) Percentage of quiescent (ICOS-/PD-1-)Th17 CD4+ T cells. AS; asymptomatic COVID-19 and S; symptomatic COVID-19 without hospitalization requirements. ns, not-significant; * *p* < 0.05.

When the Th phenotypes were analyzed according to hospitalization requirements (Figure 1), a significant difference was observed between the proportion of Th1 cells (median) in hospitalized vs. non-hospitalized COVID-19 patients: 4.5% (2.9–5.8) vs. 5.5% (3.8–7.9) (*p* = 0.034, Figure 5A). It should be noted that this decrease in the Th1 population has also been detected in the group of non-hospitalized patients who present mild symptoms when compared to asymptomatic ones: 14.4% (6–10.3) vs. 17.5% (3.7–7.1) (*p* = 0.033, Figure 6A).

Hospitalized patients showed higher proportions of early-activated Th1, Th2 and Th17 than non-hospitalized patients: 0.4% (0.2–0.6) vs. 0.3% (0.1–0.5) (*p* = 0.02, Figure 5D) for Th1, 0.5% (0.2–1) vs. 0.3% (0.1–0.5) (*p* = 0.005, Figure 5E) for Th2 and 0.3% (0.2–0.5) vs. 0.1% (0.1–0.22) (*p* = 0.002, Figure 5F) for Th17.

In addition, hospitalized patients showed higher median proportions of late-activated Th1: 1.3% (0.8–2.9) vs. 0.8% (0.5–1.9) (*p* = 0.016, Figure 5G), Th2: 0.4% (0.2–1.2) vs. 0.2% (0.2–0.4) (*p* = 0.002, Figure 5H) and Th17: 0.2% (0.1–0.4) vs. 0.1% (0.1–0.2) (*p* = 0.001, Figure 5I) compared to non-hospitalized patients.

The Th cells senescence status in hospitalized patients compared to their non-hospitalized counterparts showed increased median proportions of senescent Th17: 2.4% (1.6–3.4) vs. 1.7% (1.3–2.2) (*p* = 0.001, Figure 5L). Significant differences were not found for Th1 and Th2 senescence according to hospitalization requirements. However, higher median proportion of senescence Th1 cells were found in non-hospitalized asymptomatic patients compared to symptomatic ones: 10.4% (7.2–13.5) vs. 7.4% (5–9) (*p* = 0.033, Figure 6J). These asymptomatic patients mounted a robust Th1 response compared to healthy controls: 10.4% (7.2–13.5) vs. 4.3% (3–7.6) (*p* = 0.002, Table S3), accompanied with diminished senescence Th2 cells: 3.2% (2-5.9) vs. 11.5% (7.7–15.2) (*p* = 0.002, Table S3).

No significant results were observed when comparing the Th polarization between asymptomatic and healthy controls, hospitalization requirements and non-hospitalized patients (see Supplementary Table S3 and Figures S5 and S6, respectively).

### 3.6. Th1, Th2 and Th17 Subsets in Asymptomatic COVID-19 Patients Compared to Different Clinical Profiles

**Figure 7:**
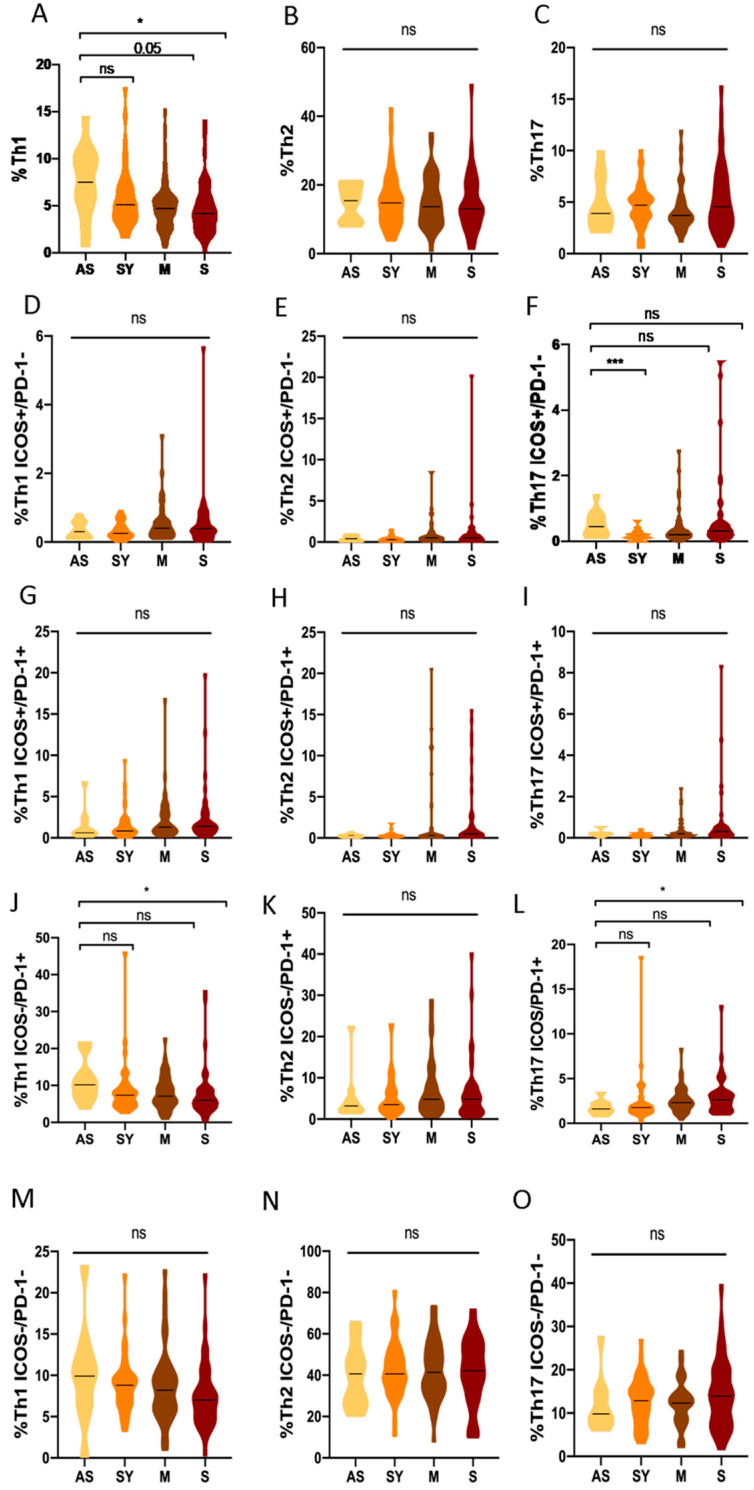
Th subsets: comparison of asymptomatic COVID-19 vs. symptomatic ones. (**A**) Total percentage of Th1 CD4+ T cells (**B**) Total parentage of Th2 CD4+ T cells (**C**) Total percentage of Th17 CD4+ T cells (**D**) Percentage of early-activated (ICOS+/PD-1-)Th1 CD4+ T cells (**E**) Percentage of early-activated (ICOS+/PD-1-)Th2 CD4+ T cells (**F**) Percentage of early-activated (ICOS+/PD-1-)Th17 CD4+ T cells (**G**) Percentage of late-activated (ICOS+/PD-1+)Th1 CD4+ T cells (**H**) Percentage of late-activated (ICOS+/PD-1+)Th2 CD4+ T cells (**I**) Percentage of late-activated (ICOS+/PD-1+)Th17 CD4+ T cells (**J**) Percentage of senescent (ICOS-/PD-1+)Th1 CD4+ T cells (**K**) Percentage of senescent (ICOS-/PD-1+)Th2 CD4+ T cells (**L**) Percentage of senescent (ICOS-/PD-1+)Th17 CD4+ T cells (**M**) Percentage of quiescent (ICOS-/PD-1-)Th1 CD4+ T cells (**N**) Percentage of quiescent (ICOS-/PD-1-)Th2 CD4+ T cells (**O**) Percentage of quiescent (ICOS-/PD-1-)Th17 CD4+ T cells. AS; asymptomatic COVID-19 patients, SY; symptomatic COVID-19 patients without hospitalization requirements, M; mild to moderate hospitalized COVID-19 patients and S; Severe COVID-19 patients. ns, not significant; * *p* < 0.05; *** *p* < 0.001.

When the differences in Th responses were analyzed according to the progression of the disease at the acute phase of the infection (Figure 1), a higher Th1 response (quantified as total percentage of Th1 cells) of asymptomatic patients was observed compared to the severe ones: the median proportion of Th1 cells was 7.5% (5.3–10.2) vs. 4.1% (2.7–5.9) (*p* = 0.015, Figure 7F).

Regarding the activation status in asymptomatic patients, we observed that they showed an increased median of senescent Th1 cells: 10.4% (7.2–13.5) vs. 6% (4–8.9) (*p* = 0.001, Figure 7J) and a diminished median of senescent Th17 cells: 1.65% (1.3–2.2) vs. 2.6% (1.6–3.4) (*p* = 0.045, Figure 7L) response compared to severe patients. There were no significant results in the Th subsets according to disease severity (Figure 7).

### 3.7. Analysis of Comorbidities in COVID-19 Patients

The association of comorbidities and hospital admission is represented in Table 3. No significant association was observed between the presence of comorbidities and the requirement for hospital admission. However, in hospitalized patients, the presence of comorbidities was associated with a higher risk of requiring ICU treatment (OR: 3.29, 95% CI: 1.09–9.95, *p* = 0.031). After the evaluation of each comorbidity separately, only obesity behaved as a significant risk factor for admission to the ICU (OR: 3.44; 95% CI: 1.10–10.83; *p* = 0.029).

### 3.8. Multivariate Analysis in COVID-19 Disease

Several different multivariate analyses that included the significant immunological and inflammatory variables obtained in a previous univariate analysis (Table 4) were performed in order to determine the relevance of the type of immune response in COVID-19 severity in relation to other significant variables associated with a poor evolution. The proportion of Th1 (OR: 0.22, 95% CI: 0.06–0.77, *p* = 0.018) in the early immune response to SARS-CoV-2 infection and higher levels of LDH (OR: 1.1, 95% CI: 1–1.1, *p* = 0.016) were identified as a protection and risk factor, respectively, for hospitalization requirements with an area under the curve ROC of 0.802 (95% CI AUC: 0.723–0.867, Table 4A).

A second multivariate analysis was performed according to the presence of severe forms of the disease. In this case, the proportion of late-activated Th2 cells (OR: 5.71, 95% CI: 1.65–19.74, *p* = 0.005) and proportion of quiescent Th17 cells (OR: 2.9, 95% CI: 1.02-8.23, *p* = 0.045) were identified as risk factors for the development of severe forms of the infection. However, the proportion of quiescent Th1 cells (OR: 0.34, 95% CI: 0.13–0.85, *p* = 0.022) was found to be a protective factor against the development of severe forms, of all which had an area under the curve ROC of 0.801 (95% CI AUC: 0.7.2–0.863, Table 4B).

A third multivariate analysis was performed to study the differences between non-hospitalized patients and those patients who required hospitalization with mild to moderate symptoms (Table 4C). The activation status of CD4+ T cells (OR: 2.58, 95% CI: 1.12–2.95, *p* = 0.026) was a significant and independent factor with an area under the curve ROC of 0.762 (95% CI AUC: 0.334–0.841).

The fourth multivariate analysis studied the differences between non-hospitalized patients and severe forms of the disease (Table 4D). Both the presence of comorbidities (OR: 3.6, 95% CI: 1.21–10.71, o = 0.021) and CD8+ T cells activation (OR: 5.91, 95% CI: 1.93–18.03, *p* = 0.002) were independent risk factors for the development of severe forms of the disease. However, the total number of lymphocytes (OR: 0.24, 95% CI: 0.07–0.73, *p* = 0.012), along with the proportion of Th1 immune response (OR: 0.17, 95% CI: 0.03–0.81, *p* = 0.025) were found to be a significant and independent protective factor, together with an area under the curve ROC of 0.844 (95% CI AUC: 0.756–0.909).

A fifth analysis was performed to compare mild to moderate patients with their severe counterparts (Table 4E). The presence of at least one comorbidity (OR: 5.083, 95% CI: 1.54–16.75, *p* = 0.007) and high levels of LDH (OR: 1, 95% CI: 1–1.01, *p* = 0.013) were significant and independent risk factors for a poor prognosis. Nonetheless, the proportion of senescent CD4+ T cells (OR: 0.11, 95% CI: 0.02–0.64, *p* = 0.014) and the proportion of quiescent Th1 (OR: 0.27, 95% CI: 0.08–0.86, *p* = 0.027) were protective factors. These results were accompanied with an area under the curve ROC of 0.86 (95% CI AUC: 0.770–0.925).

A sixth multivariate study was also performed to analyze asymptomatic COVID-19 patients and symptomatic patients who did not require hospitalization (Table 4F). The proportion of the Th1 (OR: 0.23, 95% CI: 0.05–0.96, *p* = 0.045) immune response against the SARS-CoV-2 infection was a significant and independent protective factor for the development of symptoms with an area under the curve ROC of 0.679 (95% CI AUC: 0.557–0.815).

**Figure 8:**
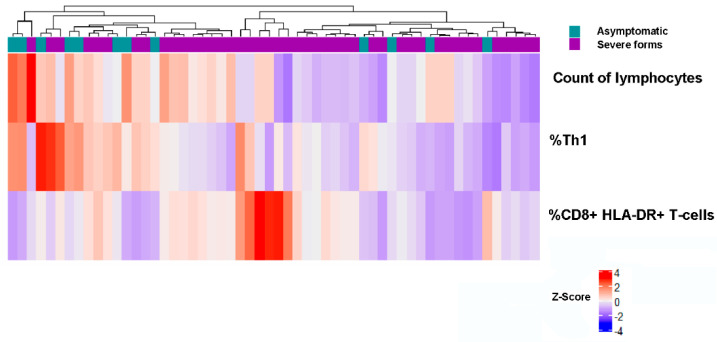
Heatmap and hierarchical clustering analysis of asymptomatic COVID-19 patients vs. COVID-19 patients with severe forms of the disease. Two different groups are represented, asymptomatic patients with higher total number of lymphocytes and robust Th1 immunity and those patients with severe forms of the disease with a higher proportion of activated CD8+ T cells.

The final and seventh analysis studied which parameters were associated with asymptomatic and severe forms of the disease (Table 4G and Figure 8). The proportion of activated CD8+ T cells (OR: 6.67, 95% CI: 1.05–45.6, *p* = 0.044) and the total proportion of %Th1 immune response (OR: 0.09, 95% CI: 0.01–0.63, *p* = 0.015) resulted in a significant and independent risk and protective factor, respectively, with an area under the curve ROC of 0.873 (95% CI AUC: 0.714–0.922).

## 4. Discussion

COVID-19 is a condition that shows a well-characterized spectrum of clinical profiles, going from asymptomatic patients to those who develop life-threatening complications, even death [41]. The latter patients are distinguished by showing alterations in their immunological profile, lymphopenia, neutrophilia or eosinophilia standing out among these [42,43,44].

As has occurred in other infectious diseases, the immune response mounted around the disease is determinant for its outcome. The presence of an effective and well-controlled adaptive immune response is sufficient for the clearance of a mild COVID-19 [45]. However, an exacerbated and persistent adaptive immune response identified by activated and exhausted phenotypes of cytotoxic T-cells with an impairment of the Th response has been associated with severe forms of the disease [32,46].

Based on a comprehensive immune profile created in this present study and the results obtained, we have been able to demonstrate that the early immune response in COVID-19 patients who require hospital admission differs from that developed by patients who do not need to be hospitalized. Interestingly, non-hospitalized COVID-19 patients established an early, effective and robust Th1 response, which behaves as an independent protective factor in the multivariate analysis of factors associated with an unfavorable disease evolution. These facts suggest a similar scenario between COVID-19 and other infective diseases like leishmaniasis, leprosy or HIV infection where the early immune response based on Th1 cells is associated with better outcomes [9,47,48,49].

Although COVID-19 is a condition that affects a wide range of ages, from childhood to old age, the most serious forms mainly affect the elderly [50,51,52]. Our results are in consonance with that fact; hospitalized infected patients were older than those patients who did not require hospital care. Likewise, the distribution of severity by sex revealed that male gender presented higher rates of hospitalization compared to females. As the severity increases, the proportion of men is higher in each group, and the biggest differences being found are in deceased patients [6,53].

In our cohort, the elevation of biomarkers associated with inflammation, such as LDH, CRP and DD, were associated with higher rates of hospitalization, confirming the relevance of inflammation in the pathophysiology and prognosis of the disease, as previously reported [54].

The T-cell profile in the early stages of the infection was different in those patients who later evolved to more complex forms of the disease and required hospital treatment. While patients who did not require hospitalization showed an immune status similar to that of healthy controls, hospitalized patients presented global lymphopenia, this being in line with previous studies where lymphocyte count had been proposed as a predictor of severity [42,55]. Likewise, these patients presented other important alterations, the phenotype and proportion of Th1 and CD8+ T-cells compartments being especially affected.

From the analysis of the T-cell profile observed in the different clinical variations, we can consider that the evolution of COVID-19 depends on the type of initial Th response. Establishment of a robust Th1 response makes it possible to effectively control viral clearance and avoid the development of complex symptoms: good prognosis COVID-19 patients showed a potent Th1 immune response compared to critically ill patients. These results are in line with other publications where the presence of higher proportions of Th1 cells was associated with mild forms of the disease [56]. The robust Th1 immune response is not only determined by the total proportion of Th1 or senescent cells. The proportion of quiescent Th1 cells could be a marker of the turnover associated with the activation and exhaustion of Th1 immunity.

A work conducted by Salehi Khesht et al. that studied the responses to SARS-CoV-2 with different immunological profiles of T-cells found a positive correlation between the severity of the disease and the Th1 response [57], which could contradict our results. However, Salehi Khesht et al. did not carry out a phenotypic study of the lymphocytic population and their study also did not indicate at what point in time of the infection the immune profiles were evaluated (acute or convalescent), so it is not possible to assess whether they mediated a rapid action against the infection or if it occurred. Furthermore, the increase in Th1 could not only be ineffective, but it also induces a polyclonal activation with the consequent pro-inflammatory scenario and severe forms of the disease [58].

The cytokines made by Th1, mainly interferon-gamma, are essential to mediate cell-mediated immunity against viruses and intracellular pathogens [59]. It has been demonstrated in our work that a global Th1 immunity is a protective factor for the development of severe forms of the disease in which hospitalization requirements are needed. A weak or absent Th1 response would imply poor ability of these pathogens to slow the disease progression [10]. When this scenario arises, alternative responses that may be essentially mediated by Th2 and Th17 are developed. In the case of COVID-19, these responses may end up being inadequate and may contribute to the immunopathology of the disease with the production of cytokines and the recruitment of immune cells to the lung [60,61].

The Th2 responses against infection mainly based on the production of antibodies [62] may not be efficient enough to eliminate the infection, since the scope of antibodies at the intracellular level is minimal [63]. Our results have shown how a prominent Th2 response results in severe forms of the disease. This fact confirms the findings in our previous work, where we described that COVID-19 patients who eventually died had established a potent Th2 response as a compensatory mechanism for an impaired Th1 response [32].

The Th17 response has been associated with autoimmune and lung inflammation [64]. Cells of the Th17 lineage present both pathogenic and protective functions in COVID-19: IL-17 recruits innate immune cells in the lungs to eliminate infection, but on the contrary, uncontrolled secretion of cytokines (IL-23/IL-17 axis) could aggravate the pathology, contributing to the cytokine released syndrome (CRS) [65,66]. We have observed a positive correlation of Th17 immunity and disease severity quantified as quiescent and senescent Th17 cells in our cohort. These results are in line with some publications, where the increase of Th17 cell activity has been observed in hospitalized COVID-19 patients, quantified by the production of IL-21 and IL-22 [27,67]. Sarmiento-Monroy et al. suggest that since cytokines released by Th17 in SARS-CoV-2 infection are hazardous in the context of CRS and ARDS, the usage of biological therapies targeting IL-23/IL-17 axis could be potentially beneficial in those scenarios [68].

We have found a negative correlation in the analysis of the absolute number of CD8+ T-cells according to disease progression. This phenomenon is analog to what occurs with CD4+ T-cells in HIV infection, where an adaptive immune response based on an efficient cell-mediated immunity makes it possible to overcome the disease without complications.

Differences were not found when both the CD4+ and CD8+ memory cells were analyzed, comparing asymptomatic to the different grades of prognosis. The instability of the proportion of the memory compartment between the different disease groups suggests long-term protection [69,70].

As many other authors have reported [42,71,72], global lymphopenia, including CD4+ and CD8+ T cells, reflects the depletion and deterioration of the immune system [73,74]. As our results have shown, CD8+ activation could play an important role in the pathogenesis link to a dysregulation of the immune system. The persistent CD8+ activation in severe patients could be translated as the last-ditch effort of the immune system to clear the infection, since the global Th1 response is absent or ineffective when associated with a pathogenic Th17 response, contrary to what has been observed in asymptomatic patients.

Since the Th1 response seems to be responsible for the correct clearance of the infection, new therapeutic strategies in this disease should be aimed at enhancing this type of response. Recent publications have shown that COVID-19 vaccines induce a bias towards Th1 immune response similar to that which we have observed in the natural infection of asymptomatic patients [75,76,77]. Likewise, the development of new vaccines and the selection of adjuvants and delivery systems should not only be oriented towards the production of antibodies but also towards ensuring that robust Th1 immunity is achieved [78,79,80,81].

This study has several limitations. The main limitation is the size of the cohort and although it is not negligible, some groups are still small when stratified according to severity. Due to the high degree of dispersion of the results, the univariate and multivariate evaluation could not be performed with raw data. Therefore, each variable needed to be transformed into ranges using a frequency analysis histogram. In the same way, the number of controls that have never had contact with viral proteins (healthy blood donors) is limited, due to the difficulties in recruiting them during the pandemic and subsequently due to the fact that the vast majority of blood donors in our environment are vaccinated against SARS-CoV-2.

## 5. Conclusions

The patients infected by SARS-CoV-2 who have a better evolution are those who establish an initial robust Th1 response against the virus, so that Th2-type responses would be associated with a more complex evolution. Thus, the determination of the type of response which predominates at the beginning of the infection could be used as a tool to discriminate patients who really require hospitalization from those who do not.

New therapies and vaccines should be based on Th1 cellular immune responses stimulation, which could rule out the development of severe forms of COVID-19-like infections.

## Figures and Tables

**Table 1:** Population characteristics of non-hospitalized and hospitalized COVID-19 patients.

Variables	Non-Hospitalized; *n* = 54	Hospitalized; *n* = 92	*p*-Value
	*n*(%)/Median (IQR)	*n*(%)/Median (IQR)	
Age	48.5 (39–63)	58.5 (46–72)	0.004
Sex (male)	29 (54%)	58 (63%)	0.038
LDH	276.5 (222.5–310)	336 (225.5–402.2)	0.001
CRP	2.9 (1.3–5.2)	6 (3.7–11)	<0.001
DD	555 (299.5–907.5)	691 (410–1414)	0.058

LDH; lactate dehydrogenase; CRP; C-Reactive protein, DD; Dimer-D.

**Table 2:** Differential T phenotype between healthy controls and COVID-19 patients.

	Healthy Control; *n* = 29		COVID-19 Cohort; *n* = 146		
Variables	Median	IQR	Median	IQR	*p*-Value
Lymphocytes	1846	1585–2129	1100	800–1500	< 0.001
CD4 count	783	664–1099	636	416–708	0.012
%CD4 Naïve	35	29.32–46.65	30.3	18.8–38.1	0.028
%CD4 CM	41	30.22–46	36.7	27.97–46.2	0.499
%CD4 EM	18.3	13.6–24.9	25.8	18.6–35.2	0.001
%CD4 TEMRA	0.7	0.3–1.92	1.5	0.5–4	0.087
%CD4 activation	4.1	3.47–5.52	6.2	3.5–9.5	0.023
%CD4 senescence	7.4	4.85–12.9	5.5	2.9–10.1	0.112
%CD4 double positive	0.8	0.5–1.17	1	0.4–2.4	0.505
CD8 count	404	285–524	296	185–477	0.022
%CD8 Naïve	27.6	17.8–41.12	13	5.5–23	<0.001
%CD8 CM	13.7	8.37–18.7	8.3	5.1–11.2	<0.001
%CD8 EM	42	26.6–49.9	48.1	40–60.2	<0.001
%CD8 TEMRA	10	7.52–24.5	20.5	11–30.5	0.003
%CD8 activation	9.5	6–12.6	18	9.1–26	<0.001
%CD8 senescence	26.2	20–35.9	38.7	18.75–50	0.024
%CD8 double positive	4.5	2.4–8.32	6	2.67–12.95	0.08

Naive (CCR7+ (CD45RA+); CM, Central memory (CCR7+/CD45RA-), EM, Effector memory (CCR7-/CD45RA-); TEMRA (CCR7-/CD45RA+), Terminal differentiated T memory cells; activated T-cells, HLA-DR+; senescent; CD57+ HLA-DR+/CD57+, double positive.

**Table 3:** Comorbidities in hospitalized vs. non-hospitalized patients (upper zone) and in those hospitalized patients who required admission to the ICU (lower zone).

Condition (All Patients)	Hospitalized (*n* = 92)		Not Hospitalized (*n* = 54)		*p* Value	OR	95% CI
Patients with comorbidities	51	(55.4%)	22	(40.7%)	0.0865		
Myocardial infarction	0	(0%)	1	(1.9%)	0.3699		
Diabetes mellitus	18	(19.6%)	7	(13%)	0.3066		
Advanzed chronical kidney disease	0	(0%)	2	(3.7%)	0.1352		
Active smokers	3	(3.3%)	4	(7.4%)	0.4239		
Former smokers	12	(13%)	3	(5.6%)	0.1718		
Obesity	16	(17.4%)	4	(7.4%)	0.1336		
Dyslipidemia	23	(25%)	9	(16.7%)	0.2416		
Hypertension	27	(29.3%)	13	(24.1%)	0.4903		
**Condition (Hospitalized Patients)**	**ICU Treated** **(*n* = 21)**		**Not ICU** **(*n* = 71)**				
Patients with comorbidities	16	(76.2%)	35	(49.3%)	0.0303	3.29	1.09–9.95
Myocardial infarction	0		0				
Diabetes mellitus	5	(23.8%)	13	(18.3%)	0.5768		
Advanzed chronical kidney disease	0		0				
Active smokers	2	(9.5%)	1	(1.4%)	0.1293		
Former smokers	3	(14.3%)	9	(12.7%)	1.0		
Obesity	7	(33.3%)	9	(12.7%)	0.0291	3.44	1.10–10.83
Dyslipidemia	7	(33.3%)	16	(22.5%)	0.3154		
Hypertension	9	(42.9%)	18	(25.4%)	0.1217		

**Table 4:** Risk factors associated with hospitalization requirements.

Variables	Univariate			Multivariate		
	OR	OR 95% CI	*p*-Value	OR	OR 95% CI	*p*-Value
(A) NH vs. H						
Sex (male)	2.13	1.07–4.22	0.03	1.3	0.54–3.11	0.544
Age	1.03	1–1.04	0.006	1.02	0.9–1.04	0.077
Lymphocytes	0.3	0.13–0.65	0.003	0.47	0.18–1.23	0.126
%CD4 activation	2.87	1.3–5.94	0.005	2.53	0.94–6.82	0.066
%CD8 activation	2.42	1.14–5.12	0.02	1.41	0.48–4.07	0.526
%Th1	0.3	0.11–0.78	0.014	0.18	0.04–0.75	0.018
LDH	1	1–1.1	0.006	1.1	1–1.1	0.016
CRP	1.1	1–1.14	0.032	1	0.93–1.07	0.956
Area Under the ROC Curve					0.802	(0.723–0.867)
(B) NS vs. S						
Age	1.02	1–1.04	0.023	1.02	0.99–1.05	0.069
Comorbidity	2.94	1.39–6.2	0.004	2.57	0.9–7.35	0.076
Obesity	3.44	1.31–9.05	0.012	2.08	0.67–6.42	0.199
Lymphocytes	0.4	0.19–0.86	0.019	0.49	0.2–1.23	0.131
%Th1 quiescence	0.37	0.17–0.80	0.012	0.34	0.13–0.85	0.022
%Th2 late-activation	3.41	1.24–9.38	0.017	5.71	1.65–19.74	0.005
%Th17 quiescence	2.3	1.01–5.24	0.046	2.9	1.02–8.23	0.045
Area Under the ROC Curve					0.801	(0.727–0.863)
(C) NH vs. M						
Age	1.01	0.99–1.04	0.072	1.01	0.99–1.04	0.199
Lymphocytes	0.37	0.15– 0.89	0.026	0.94	0.28–2.83	0.086
CD4 count	0.44	0.26–0.75	0.002	0.62	0.31–1.25	0.185
%CD4 activation	2.8	1.3–6.08	0.008	2.58	1.12–2.95	0.026
%Th1	0.36	0.12–1.12	0.078	0.32	0.8–1.23	0.099
Area Under the ROC Curve					0.762	(0.334–0.841)
(D) NH vs. S						
Comorbidity	3.11	1.35–7.18	0.007	3.6	1.21–10.71	0.021
Obesity	4.16	1.22–14.19	0.022	4.48	0.81–25	0.086
Lymphocytes	0.23	0.089–0.63	0.004	0.24	0.07–0.73	0.012
%CD8 activation	4.14	1.75–9.83	0.001	5.91	1.93–18.03	0.0018
%Th1	0.23	0.06–0.87	0.03	0.17	0.03–0.81	0.025
%Th17 early-activation	6.79	0.76–60.5	0.085	1.53	0.13–16.93	0.725
Area Under the ROC Curve					0.844	(0.756–0.909)
(E) M vs. S						
Comrbidity	2.75	1.17–6.46	0.019	5.08	1.54–16.75	0.007
%CD4 senescence	0.33	0.09–1.14	0.082	0.11	0.02–0.64	0.014
%CD8 activation	2.89	1.23–6.79	0.014	2.53	0.54–11.92	0.237
%CD8 double positive	1.09	1.03–1.16	0.004	1.12	0.99–1.27	0.052
%Th1 quiescence	0.43	0.18–1.03	0.061	0.27	0.08–0.86	0.027
LDH	1	1–1.01	0.008	1	1–1.01	0.013
Area Under the ROC Curve					0.86	(0.770–0.925)
(F) A vs. SY						
%CD4 CD57+	0.23	0.03–1.33	0.12	0.31	0.04–2.07	0.231
%Th1	0.2	0.049–0.8	0.023	0.23	0.05–0.96	0.045
Area Under the ROC Curve					0.697	(0.557–0.815)
(G) A vs. S						
Lymphocytes	0.18	0.04–0.81	0.025	0.38	0.07–1.95	0.247
%CD8 activation	6.57	1.28–33.61	0.023	6.67	1.05–45.6	0.044
%Th1	0.07	0.01–0.37	0.002	0.09	0.01–0.63	0.015
Area Under the ROC Curve					0.837	(0.714–0.922)

Activated T-cells, HLA-DR+; senescence; CD57+ and ICOS-/PD-1+; double positive, HLA-DR+/CD57+; early-activation cells, ICOS+/PD-1-; late-activation cells, ICOS+/PD-1 quiescence, ICOS-/PD-1-; LDH, lactate dehydrogenase; CRP, C-Reactive protein.

## Data Availability

The data that support the findings of this study are available from the corresponding author, upon reasonable request.

## Supplementary Materials

The following are available online at https://www.mdpi.com/article/10.3390/biomedicines10020296/s1, Figure S1: Lymphocytic phenotype in asymptomatic and symptomatic COVID-19 without hospitalization requirements, Figure S2: CD4+ subsets: comparison of asymptomatic COVID-19 patients according to disease severity, Figure S3: CD8+ subsets: comparison of asymptomatic COVID-19 patients vs. symptomatic ones, Table S1: Antigenic specificity of anti-SARS-CoV-2 depending on hospitalization requirements, Table S2: Differential Th subsets between healthy controls and COVID-19 patients, Table S3: Differential immunological profile between asymptomatic COVID-19 patients and healthy controls.
