# Supplementary material for: An Early Th1 Response Is a Key Factor for a Favorable COVID-19 Evolution
Source: Biomedicines. 2022 Jan 27;10(2):296. doi: 10.3390/biomedicines10020296 (PMC8869678; doi:10.3390/biomedicines10020296)
Supplement: Supplementary file 1 [file biomedicines-10-00296-s001.zip › biomedicines-1531923-supplementary.pdf]

Table S1. Antigenic specificity of anti-SARS-CoV-2 depending on hospitalization requirements.

Variables	Non-hospitalized; N=53 Number (%)	Hospitalized; N=92 Number (%)	p-value
Serology	27 (51%)	48 (52%)	0.076
S1 domain	20 (38%)	37 (40%)	0.73
S2 domain	17 (32%)	20 (22%)	0.783
RBD domain	23 (43%)	39 (42%)	0.949
Ncap protein	20 (38%)	41 (45%)	0.39

Table S2. Differential Th subsets between healthy controls and COVID-19 patients.

	**Healthy controls; N=29**		**COVID-19 cohort; N=146**		
Variables	Median	IQR	Median	IQR	p-value
%Th1	6.4	4.95-8.2	4.8	3.3-6.5	0.01
%Th2	16.8	11.37-20.9	13.85	9.3-19.7	0.229
%Th17	6.95	3.08-10.57	4	3.1-5.7	0.018
%Th1 early-activation	1.7	0.7-2.1	0.3	0.2-0.6	< 0.001
%Th2 early-activation	2.81	1.32-3.6	0.4	0.2-0.8	< 0.001
%Th17 early-activation	1.2	0.62-1.8	0.2	0.1-0.4	< 0.001
%Th1 late-activation	1.48	0.96-2.36	1.2	0.6-2.46	0.254
%Th2 late-activation	1.2	1.45-1.89	0.3	0.2-0.5	< 0.001
%Th17 late-activation	1.44	0.57-2.1	0.2	0.1-0.3	< 0.001
%Th1 senescence	4.27	3.03-7.65	7	4.85-9.785	0.008
%Th2 senescence	11.5	7.772-15.2	4.3	2.1-7.65	< 0.001
%Th17 senescence	3.21	1.55-7	2.1	1.4-3.1	0.036
%Th1 quiescence	9.76	6.62-11.77	7.91	5.9-10.1	0.097
%Th2 quiescence	39.16	27.22-42.55	41.4	31.7-52.52	0.051
%Th17 quiescence	14.35	4.98-19.3	12.6	8-15.5	0.941

Early-activation cells , ICOS+/PD-1-; late-activation cells, ICOS+/PD-1+; senescence, ICOS-/PD-1+;quiescence, ICOS-/PD-1-

Table S3. Differential immunological profile between asymptomatic COVID-19 patients and healthy controls.

Variables	Asymptomatic; N=12		Healthy controls; N=29		
	Median	IQR	Median	IQR	p-value
Lymphocytes	1500	1200 - 2050	1846	1585 - 2129	0.121
CD4 count	793.5	604 - 1307	783	644 - 1099	0.908
%CD4 Naïve	27.05	15.65 - 45	35	29.32 - 43.65	0.202
%CD4 CM	38.25	30.5 - 44.5	41	30.22 - 46	0.528
%CD4 EM	27.15	17.25 - 35.95	18.3	13.57 - 24.87	0.108
%CD4 TEMRA	2.3	0.85 - 4.56	0.7	0.3 - 1.92	0.062
%CD4 activation	6.25	2.95 - 8.8	4.1	3.47 - 5.52	0.785
%CD4 senescence	4.5	2.95 - 16.1	7.4	4.85 - 12.9	0.406
%CD4 double positive	0.6	0.3 - 3.65	0.8	0.5 - 1.17	0.709
CD8 count	504	383. - 7878	404	285- 524	0.051
%CD8 Naïve	11.95	3.8 - 24.55	27.6	17.8 - 41.12	0.005
%CD8 CM	5.85	3.25 - 7.6	13.7	8.37 - 18.7	<0.001
%CD8 EM	47.7	37.2 - 62.05	42	26.55 - 49.85	0.083
%CD8 TEMRA	24.95	17.05 - 33.55	10	7.52 - 24.5	0.03
%CD8 activation	16.45	8.75 - 22.2	9.5	6.05- 12.6	0.083
%CD8 senescence	45.6	21.1 - 63.05	26.2	20 - 35.85	0.048
%CD8 double positive	7.2	3.3 - 15.85	4.5	2.4 - 8.32	0.169
%Th1	7.5	5.3 - 10.2000	6.7	4.95 - 8.2	0.653
%Th2	15	9.2 - 19.35	16.8	11.37 - 20.9	0.454
%Th17	3.75	2.3 - 6.4	6.95	3.08 - 10.57	0.092
%Th1 early-activation	0.25	0.1 - 0.55	1.17	0.7- 2.06	< 0.001
%Th2 early-activation	0.45	0.2 - 0.75	2.81	1.35 - 3.6	< 0.001
%Th17 early-activation	0.15	0.1 - 0.4	1.19	0.62 - 1.79	< 0.001
%Th1 late-activation	0.65	0.5-1.95	1.48	0.96 - 2.36	0.066
%Th2 late-activation	0.3	0.2-0.35	1.49	1.14 - 1.89	< 0.001
%Th17 late-activation	0.2	0.1-0.2	1.44	0.57 - 2.1	< 0.001
%Th1 senescence	10.35	4.2-13.5	4.27	3.03 - 7.65	0.002
%Th2 senescence	3.2	1.95-5.9	11.5	7.72- 15.20	0.002
%Th17 senescence	1.65	1.25-2.25	3.21	1.55 - 6.98	0.051
%Th1 quiescence	9.85	5.95-11.95	9.76	6.62 - 11.77	0.881
%Th2 quiescence	9.85	7.6-14.2	14.35	4.98 - 19.3	0.697
%Th17 quiescence	35.35	14.8-48.15	35.9	27.22 - 42.55	0.736

Naive (CCR7+ (CD45RA+); CM, Central memory (CCR7+/CD45RA-), EM, Effector memory (CCR7-/CD45RA-); TEMRA (CCR7-/CD45RA+), Terminal differentiated T memory cells; activated T-cells, HLA-DR+; senescence; CD57+; double positive, HLA-DR+/CD57+; early-activation cells , ICOS+/PD-1-; late-activation cells, ICOS+/PD-1+; senescence, ICOS-/PD-1+;quiescence, ICOS-/PD-1-.

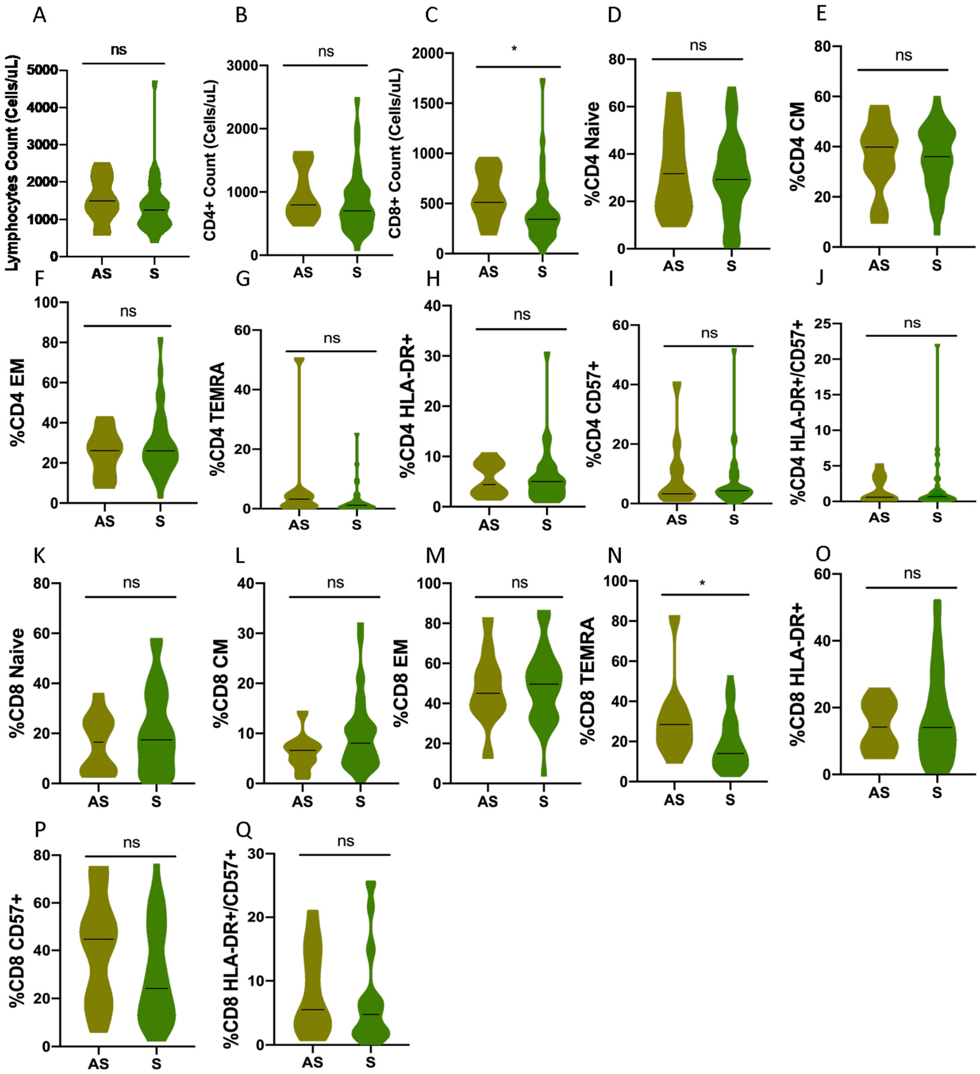

Figure S1. Lymphocytic phenotype in asymptomatic and symptomatic COVID-19 without hospitalization requirements. A) Lymphocyte count (cells/ul) B) Number of CD4+ (cells/ul) T cells C) Number of CD8+ (cells/ul) T cells D) Percentage of naive (CCR7+/CD45RA+) CD4+ T cells E) Percentage of CM (central memory, CCR7+/CD45RA-) CD4+ T cells F) Percentage of EM (effector memory, CCR7-/CD45RA-) CD4+ T cells G) Percentage of TEMRA (Terminal differentiated T memory cells ,CCR7-/CD45RA+) CD4+ T cells H) Percentage of activated (HLA-DR+) CD4+ T cells I) Percentage of senescent (CD57+) CD4+ T cells J) Percentage of double positive (HLA-DR+/CD57+) CD4+ T cells K) Percentage of naive (CCR7+/CD45RA+) CD8+ T cells L) Percentage of CM (central memory, CCR7+/CD45RA-) CD8+ T cells M) Percentage of EM (effector memory, CCR7-/CD45RA-) CD8+ T cells N) Percentage of TEMRA (Terminal differentiated T memory cells ,CCR7-/CD45RA+) CD8+ T cells O) Percentage of activated (HLA-DR+) CD8+ T cells P) Percentage of senescent (CD57+) CD8+ T cells Q) Percentage of double positive (HLA-DR+/CD57+) CD8+ T cells. AS; asymptomatic COVID-19 and S; symptomatic COVID-19 without hospitalization requirements. ns, not-significant; * p 0.05.

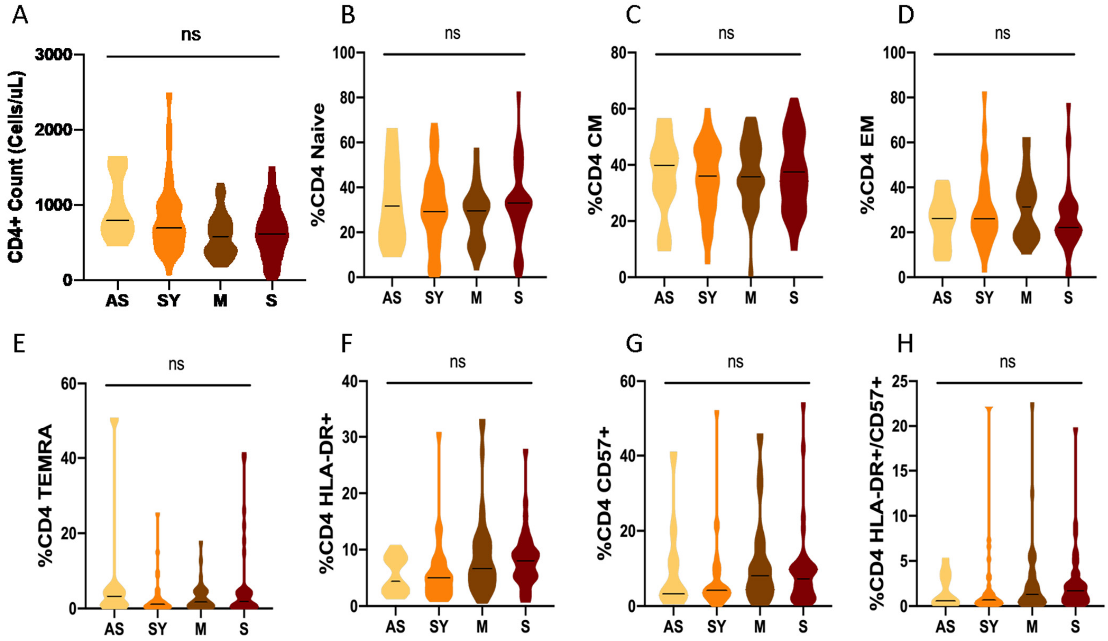

Figure S2. CD4+ subsets: comparison of asymptomatic COVID-19 patients according to disease severity. A) Number of CD4+ (cells/ul) T cells B) Percentage of naive (CCR7+/CD45RA+) CD4+ T cells C) Percentage of CM (central memory, CCR7+/CD45RA-) CD4+ T cells D) Percentage of EM (effector memory, CCR7-/CD45RA-) CD4+ T cells E) Percentage of TEMRA (Terminal differentiated T memory cells ,CCR7-/CD45RA+) CD4+ T cells F) Percentage of activated (HLA-DR+) CD4+ T cells G) Percentage of senescent (CD57+) CD4+ T cells H) Percentage of double positive (HLA-DR+/CD57+) CD4+ T cells. AS; asymptomatic COVID-19 patients, SY; symptomatic COVID-19 patients without hospitalization requirements, M; mild to moderate hospitalized COVID-19 patients and S; Severe COVID-19 patients. ns, not-significant.

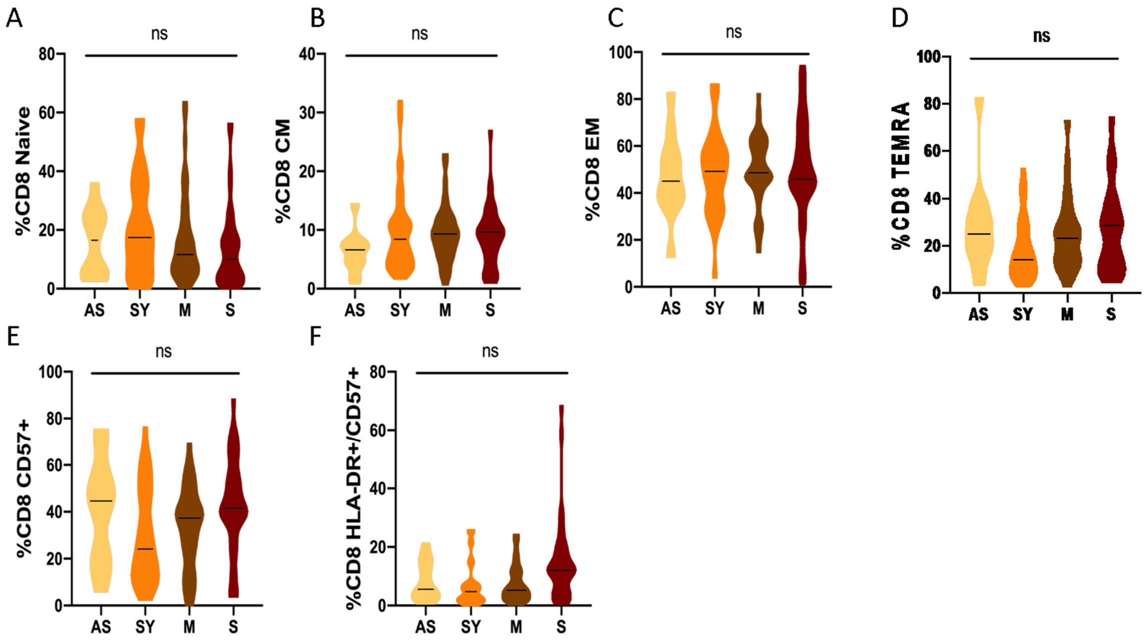

Figure S3. CD8+ subsets: comparison of asymptomatic COVID-19 patients vs. symptomatic ones. A) Percentage of naive (CCR7+/CD45RA+) CD8+ T cells B) Percentage of CM (central memory, CCR7+/CD45RA-) CD8+ T cells C) Percentage of EM (effector memory, CCR7-/CD45RA-) CD8+ T cells D) Percentage of TEMRA (Terminal differentiated T memory cells, CCR7-/CD45RA+) CD8+ T cells E) Percentage of senescent (CD57+) CD8+ T cells F) Percentage of double positive (HLA-DR+/CD57+) CD8+ T. AS; asymptomatic COVID-19 patients, SY; symptomatic COVID-19 patients without hospitalization requirements, M; mild to moderate hospitalized COVID-19 patients and S; Severe COVID-19 patients. ns, not-significant.
